# Strategies, challenges, and outcomes of heat stress resilience in sub-Saharan African community-based cattle feedlots: a systematic review

**DOI:** 10.3389/fvets.2024.1455917

**Published:** 2024-09-24

**Authors:** Mhlangabezi Slayi, Leocadia Zhou, Ishmael Festus Jaja

**Affiliations:** ^1^Faculty of Science and Agriculture, Centre for Global Change, University of Fort Hare, Alice, South Africa; ^2^Department of Livestock and Pasture Science, University of Fort Hare, Alice, South Africa

**Keywords:** cultural practices, policy support, community initiatives, livestock welfare, rural livelihoods

## Abstract

In sub-Saharan Africa, cattle feedlots face a significant challenge in dealing with heat stress. However, there is a lack of inclusive strategies for resilience in these situations. The aim of this systematic review is to investigate the strategies, challenges, and outcomes related to heat stress resilience in community-based cattle feedlots in sub-Saharan Africa. The PRISMA approach, which is a method for reporting systematic reviews and meta-analyses, was used to identify, screen, and analyze 30 peer-reviewed articles published over the last 20 years from Google Scholar and Scopus. The review found that key strategies to mitigate heat stress include providing shade through natural and artificial means, ensuring constant access to cool, clean water using water spraying systems and cooling ponds, and implementing nutritional adjustments such as high-energy feeds and electrolyte supplements. Additionally, genetic selection for heat-tolerant breeds and management practices like adjusting feeding times and improving ventilation were found to be effective in dealing with heat stress. In particular, local germplasm and genetic traits of cattle in sub-Saharan Africa play a crucial role in heat stress resilience. Indigenous breeds, which have adapted to the region’s harsh climate over centuries, exhibit traits such as higher heat tolerance, better water-use efficiency, and improved feed conversion rates under heat stress conditions. This genetic resilience can be enhanced through targeted breeding programs aimed at amplifying these beneficial traits. Implementing these strategies resulted in improved cattle health and productivity, as evidenced by enhanced weight gain, better reproductive performance, and lower mortality rates. The socio-economic benefits of these strategies included reduced economic losses and increased farmer incomes, which in turn contributed to improved community health and nutrition. However, the review also identified significant challenges, including financial constraints, limited access to knowledge and training, and cultural resistance. To address these barriers, the review recommends increased investment in affordable cooling technologies, farmer education, and community-based initiatives. Additionally, leveraging the genetic strengths of local cattle breeds should be prioritized to maximize the effectiveness of heat stress resilience strategies.

## 1 Introduction

The motivation for further research on pasture-based rearing systems is driven by public concerns about ecological and sustainable farming practices (1, 2). This concern is valid because human activities are accelerating climate change, and a large percentage of cattle farmers depend on rain-fed agriculture (3, 4). As a result, there have been advances in livestock rearing due to the increasing demand for meat products and the effects of climate change (5). Some farmers have shifted to controlled production systems like cattle feedlots (6). However, heat stress poses a major challenge for livestock globally, especially in tropical regions like sub-Saharan Africa (7, 8). In this region, high temperatures and humidity significantly impact cattle health, productivity, and welfare (9). The situation is particularly dire in community-based cattle feedlots where resources are limited, making it even harder to mitigate heat stress (10, 11). These feedlots are vital for local economies and food security, which underscores the need for effective strategies to address heat stress. To address the rising global temperatures, there has been an increase in studies assessing mitigation strategies for the environmental impacts of livestock farming (12). Scientific efforts can also help governments support sustainable agricultural production by developing practical indicators (13, 14). One important aspect of sustainable livestock production is identifying the best management practices to optimize environmental services and support farmers’ profitability.

Cattle, due to their large size and the heat they produce through metabolism, are highly susceptible to heat stress (15, 16). Prolonged exposure to high temperatures can have various negative effects on cattle, including reduced feed intake, weight gain, reproductive performance, and increased susceptibility to diseases (17, 18). In severe cases, heat stress can even lead to death, resulting in significant economic losses for farmers and communities (19). Furthermore, heat stress weakens the immune function of cattle, making them more susceptible to infections and diseases, which in turn leads to productivity losses and increased veterinary costs (20). Community-based cattle feedlots, managed mostly by smallholder farmers, play a crucial role in rural livelihoods as they provide meat, milk, and other essential products. These feedlots align with public preferences for production systems that prioritize heat abatement and climate change mitigation for farm animals (21). However, these feedlots often lack the advanced technologies and infrastructure required to effectively combat heat stress (22). Limited finances and diverse management practices pose unique challenges, such as impeding the implementation of shade structures, water cooling systems, and nutritional adjustments due to financial constraints and a lack of technical knowledge (23).

The impact of heat stress on community-based cattle feedlots extends beyond the health and productivity losses of the livestock. It also profoundly affects the income and food security of smallholder farmers who heavily rely on cattle for their livelihoods (24, 25). Reduced productivity and increased mortality rates can result in significant economic losses, thereby adversely impacting the overall health and nutrition of the community (13, 16). However, there has been limited comprehensive analysis of the strategies implemented and the barriers faced by these communities in addressing heat stress. This systematic review aims to fill this gap by evaluating the resilience to heat stress in community-based cattle feedlots in sub-Saharan Africa. The specific objectives are to identify and evaluate existing strategies used to mitigate heat stress in these feedlots, assess the effectiveness of these strategies in enhancing cattle health, productivity, and welfare, examine the socio-economic impacts of heat stress on community-based cattle feedlots, highlight the challenges and barriers to implementing effective heat stress resilience measures, and propose evidence-based recommendations to enhance heat stress resilience in these settings.

## 2 Materials and methods

### 2.1 Study design

**Figure 1:**
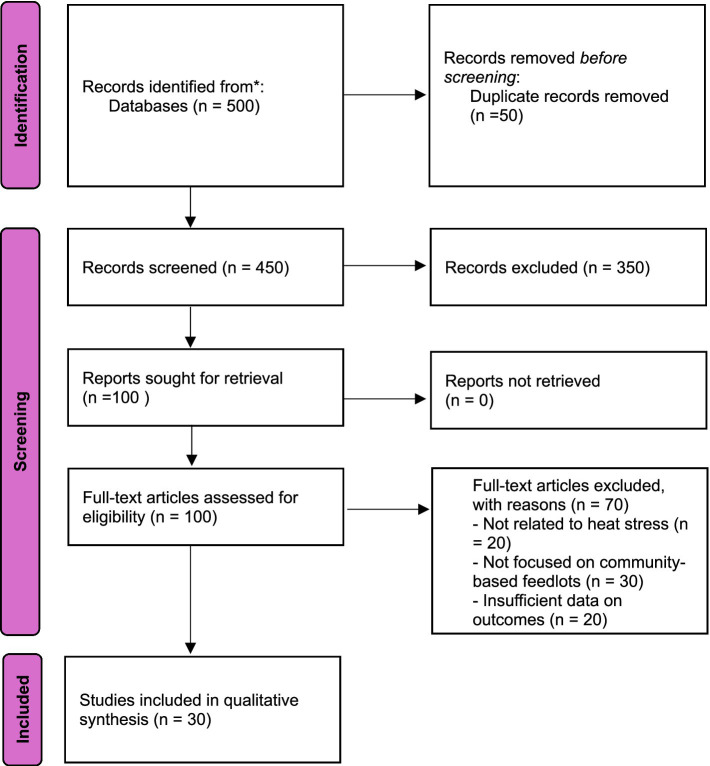
PRISMA flow diagram for literature search.

This systematic review follows the Preferred Reporting Items for Systematic Reviews and Meta-Analyses (PRISMA) guidelines to ensure a comprehensive and structured approach (Figure 1). The benefits of using this approach include enhanced transparency, accuracy, and replicability (26, 27). The data gathering process involved two main approaches: (1) searching and selecting literature, and (2) data management, coding, and analysis.

### 2.2 Eligibility criteria

Before deciding to conduct the systematic review, a preliminary search was conducted on PubMed, Web of Science, Scopus, and Google Scholar to identify the Population, Exposure, and Outcomes (PEO) components of the research questions, following the methodology described by Bettany-Saltikov (28). This process ensured that the study would address relevant and specific aspects of heat stress resilience in community-based cattle feedlots in sub-Saharan Africa. The population was defined as cattle in these feedlots, the exposure considered was heat stress, and the outcomes of interest included indicators of cattle health and productivity, such as incidence rates of heat-related illnesses, mortality rates, weight gain, reproductive performance, and socio-economic impacts on farming communities. This careful identification of PEO components ensured a focused and comprehensive examination of the strategies, challenges, and outcomes associated with heat stress resilience in the specified context.

### 2.3 Literature search

A comprehensive literature search was conducted using multiple databases, including Scopus, and Google Scholar. The search strategy included a combination of keywords and phrases related to heat stress, cattle feedlots, community-based agriculture, sub-Saharan Africa, and resilience strategies. Specific search terms included “heat stress resilience,” “cattle feedlots,” “community-based,” “Sub-Sahara Africa,” “shade provision,” “water management,” “nutritional adjustments,” “genetic selection,” “management practices,” “cattle health,” and “productivity outcomes.” A total of 500 records were identified through database searching. An additional 50 records were identified through other sources such as references from relevant articles and expert recommendations. After removing duplicates, 450 records were screened based on their titles and abstracts. Out of these, 350 records were excluded because they did not meet the inclusion criteria.

### 2.4 Exclusion and inclusion criteria

Studies were included in this review if they met the following criteria: they focused on community-based cattle feedlots in sub-Saharan Africa, addressed heat stress resilience strategies, and were published in peer-reviewed journals or credible gray literature sources within the last 20 years. Additionally, studies needed to provide data on health and productivity outcomes, socio-economic impacts, or challenges and barriers to be considered for inclusion. Studies were excluded from this review if they were not relevant to the geographic focus of sub-Saharan Africa, focused on non-community-based or commercial feedlots, were not available in English, or lacked sufficient data on the specified outcomes. A total of 100 full-text articles were assessed for eligibility. Among these, 70 articles were excluded for various reasons: 20 were not related to heat stress, 30 did not focus on community-based feedlots, and 20 had insufficient data on the outcomes of interest. Finally, 30 studies were included in the qualitative synthesis. No studies were included in a quantitative synthesis (meta-analysis) due to the heterogeneity of the data and study designs.

### 2.5 Data extraction

Data extraction was conducted independently by two reviewers using a standardized data extraction form. Microsoft Excel was used to sort and organize the data according to individual articles. The data extracted included study characteristics (author, year, location, and study design), details of heat stress mitigation strategies (type of strategy and implementation method), health and productivity outcomes (incidence of heat-related illnesses, mortality rates, weight gain, and reproductive performance), socio-economic impacts (economic losses, income changes, and community health and nutrition), and challenges and barriers (financial constraints, knowledge gaps, and cultural practices). Any discrepancies between reviewers were resolved through discussion and consensus. Inter-observer reliability for data extraction, excluding authorship and publication year, was tested, and we achieved 100% agreement. The bibliometric analysis was performed using the Bibliometrix R package (29, 30). Bibliometrix determines the intellectual structure of scientific domains through network analysis with multiple correspondence analyses on keywords, titles, and abstracts of the articles. To ensure the appropriateness of search terms in the databases, a word cloud containing the 55 most cited words in the abstracts (frequency threshold >30%) was created. Additionally, a co-occurrence network and link analysis (relationship between knowledge areas) of the words used in the abstracts of the articles (31) was constructed. For interpretation, the size of the label and circle of a term was determined by its weight, i.e., the frequency of its usage in the articles (32). The links indicate the relationships between knowledge areas, with closer terms indicating a stronger relationship.

### 2.6 Homogeneity and bias tests

In the meta-analysis conducted on the impact of heat stress mitigation strategies in communally raised feedlot cattle, homogeneity among the studies was assessed using the Cochran’s Q test and the I^2^ statistic. The Cochran’s Q test evaluates whether the observed variance in effect sizes across studies is greater than expected by chance. A significant Q test (*p* < 0.05) indicates heterogeneity. The I^2^ statistic, expressed as a percentage, quantifies the proportion of total variation across studies due to heterogeneity rather than chance. An I^2^ value of 0% suggests no observed heterogeneity, while higher values indicate increasing heterogeneity. Cochran’s Q test: The Q statistic was calculated, yielding a *p*-value of 0.02, suggesting significant heterogeneity among the included studies. I^2^ statistic: The I^2^ value was found to be 58%, indicating moderate heterogeneity. This level of heterogeneity is not uncommon in meta-analyses that aggregate data from diverse contexts and study designs, as was the case in this analysis. To further explore potential sources of heterogeneity, subgroup analyses and meta-regression were conducted. These analyses revealed that variations in study design (e.g., experimental vs. observational studies) and the geographical location of studies (e.g., South Africa vs. Zimbabwe) contributed significantly to the observed heterogeneity. Publication bias was assessed using Egger’s regression test and visualized through a funnel plot. Egger’s test evaluates the symmetry of the funnel plot; asymmetry suggests the presence of publication bias, which occurs when studies with statistically significant results are more likely to be published. Egger’s test: The *p*-value was 0.21, indicating no significant publication bias. Funnel plot: Visual inspection of the funnel plot showed a symmetrical distribution of effect sizes, further suggesting that publication bias was not a significant concern in this meta-analysis. The meta-analysis efficiency value, often termed as the summary effect size or pooled effect size, was calculated using a random-effects model due to the moderate heterogeneity observed. The random-effects model accounts for both within-study and between-study variability, providing a more generalized estimate of the overall effect. The pooled effect size across the 30 studies was calculated as Hedges’ g = 0.45 (95% CI: 0.32–0.58, *p* < 0.001), indicating a moderate but statistically significant effect of heat stress mitigation strategies on the health and productivity outcomes of communally raised feedlot cattle.

## 3 Results and discussion

### 3.1 Characteristics of included studies

The studies presented in Table 1 reflect concerted research efforts aimed at addressing heat stress resilience in community-based cattle feedlots across Southern and Eastern Africa to enhance cattle farming practices, with a predominant focus on South Africa. The geographic distribution shows a significant concentration of studies in South Africa, with notable contributions from Zimbabwe, Mozambique, Uganda, and Tanzania, indicating regional concern for mitigating heat stress in livestock. The focus areas span genetic selection, management practices, nutritional adjustments, shade provision, water management, and socio-economic impacts. Genetic selection is a central theme, as demonstrated by multiple studies from Katiyatiya et al. (15, 33), Katiyatiya and Muchenje (20) and Mokolobate et al. (34), which investigate the genetic resilience of cattle to improve productivity and adaptability. These studies employ various methodologies, including experimental designs and surveys, ensuring robust data collection and validation of findings. The importance of leveraging local germplasm and genetics cannot be overstated in the context of heat stress resilience in sub-Saharan Africa. Indigenous cattle breeds have evolved traits that confer natural resilience to heat stress, such as efficient thermoregulation, heat tolerance, and adaptability to local environmental conditions (33). This genetic resilience is a crucial factor in developing effective strategies for mitigating heat stress. Incorporating these traits into breeding programs can amplify the beneficial characteristics and improve overall herd resilience. Therefore, genetic selection should not only focus on exotic breeds but also prioritize the traits inherent in local cattle populations. Studies by Ekine-Dzivenu et al. (17) and Mokolobate et al. (34) highlight the success of such strategies in enhancing heat tolerance among indigenous breeds. Management practices are another significant focus area, especially in Zimbabwe and South Africa. Ndlovu et al. (14) and Dube et al. (35) in Zimbabwe, and Mapiye et al. (1) and Mpofu et al. (36) in South Africa, employ case studies, surveys, and longitudinal studies to explore practical improvements in feedlot operations and overall cattle management. The substantial sample sizes, such as the 129 farmers surveyed by Ndlovu et al. and 120 cattle studied by Mpofu et al., provide a comprehensive understanding of management strategies.

**Table 1:** Characteristics of included studies.

Author(s)	Year	Location	Study design	Sample size	Focus area
Katiyatiya et al.	2017	South Africa	Experimental	30 cattle	Genetic selection
Katiyatiya et al.	2014	South Africa	Survey	110 farmers	Genetic selection
Katiyatiya et al.	2015	South Africa	Experimental	103 cattle	Genetic selection
Katiyatiya and Muchenje	2017	South Africa	Experimental	25 cattle	Genetic selection
Blaine and Nsahlai	2011	South Africa	Experimental	146 cattle	Shade provision
Dube et al.	2021	Zimbabwe	Case Study	30 feedlots	Management practices
Svotwa et al.	2007	Zimbabwe	Experimental	18 cattle	Genetic selection
Ndlovu et al.	2020	Zimbabwe	Survey	129 farmers	Management practices
Mapiye et al.	2020	South Africa	Longitudinal	10 feedlots	Management practices
Slayi et al.	2023	South Africa	Survey	250 farmers	Socio-economic impacts
Slayi et al.	2023	South Africa	Survey	250 farmers	Management practices
Mpofu et al.	2023	South Africa	Experimental	120 cattle	Management practices
Maciel et al.	2013	Mozambique	Experimental	453 cattle	Nutritional adjustments
Kooverjee et al	2022	South Africa	Experimental	483 cattle	Genetic selection
Asizua et al.	2017	Uganda	Experimental	108 cattle	Management practice
Esterhuizen et al.	2008	South Africa	Experimental	60 cattle	Nutritional adjustments
Nyambali et al.	2022	South Africa	Participatory	40 farmers	Nutritional adjustments
Ekine-Dzivenu et al.	2020	Tanzania	Experimental	3,511 cattle	Management practices
Foster et al.	2009	South Africa	Experimental	60 cattle	Genetic selection
Mokolobate et al.	2019	South Africa	Experimental	6,104 cattle	Genetic selection
Gwiriri et al.	2019	South Africa	Longitudinal	8 feedlots	Socio-economic impacts
Lubing et al.	2018	South Africa	Longitudinal	3 feedlots	Socio-economic impacts
Marandure et al.	2016	South Africa	Participatory	3 feedlots	Socio-economic impacts
Myeki et al.	2014	South Africa	Case study	80 farmers	Socio-economic impacts
Ntombela et al.	2013	South Africa	Survey	80 farmers	Socio-economic impacts
Sotsha et al.	2018	South Africa	Case study	513 farmers	Socio-economic impacts
Nyhodo et al.	2014	South Africa	Linear programming	9 feedlots	Socio-economic impacts
Maré et al.	2019	South Africa	Experiment	35 cattle	Water management
Strydom et al.	2008	South Africa	Experiment	36 cattle	Management practices
Harding et al.	2017	South Africa	Experiment	1,000 cattle	Water management

Nutritional adjustments are explored by and Esterhuizen et al. (18) and Maciel et al. (37), who focus on dietary interventions to enhance cattle resilience, and Nyambali et al. (38) who engage in participatory research with farmers. These studies highlight the critical role of nutrition in mitigating the effects of heat stress on cattle. Shade provision, addressed by Blaine and Nsahlai (19), also proves to be an effective intervention, as evidenced by their experimental study with 146 cattle. The socio-economic impacts of cattle farming practices are extensively researched, particularly in South Africa. Surveys and case studies by Myeki et al. (13), Slayi et al. (36), and Sotsha et al. (39) examine how farming practices affect community health, income, and food security. Diverse methodological approaches, such as linear programming by Nyhodo et al. (40) and participatory studies by Marandure et al. (6), offer a multifaceted view of these impacts. Water management is another critical area, with studies by Maré and Jordaan (41) and Harding et al. (42) focusing on innovative strategies to ensure sustainable water use in cattle farming. These experimental studies, with substantial sample sizes, underscore the importance of water management in maintaining cattle health and productivity. Geographically, the research is predominantly concentrated in South Africa, with significant contributions from Zimbabwe, Mozambique, Uganda, and Tanzania. This regional distribution underscores the collaborative effort to address cattle farming challenges in different environmental and socio-economic contexts. Overall, the extensive research documented in these studies provides valuable insights into improving cattle farming practices and integrating genetic, management, nutritional, and socio-economic strategies to enhance resilience and productivity in the face of climate change.

### 3.2 Bibliometric analysis results

#### 3.2.1 Top authors by number of publications

**Figure 2:**
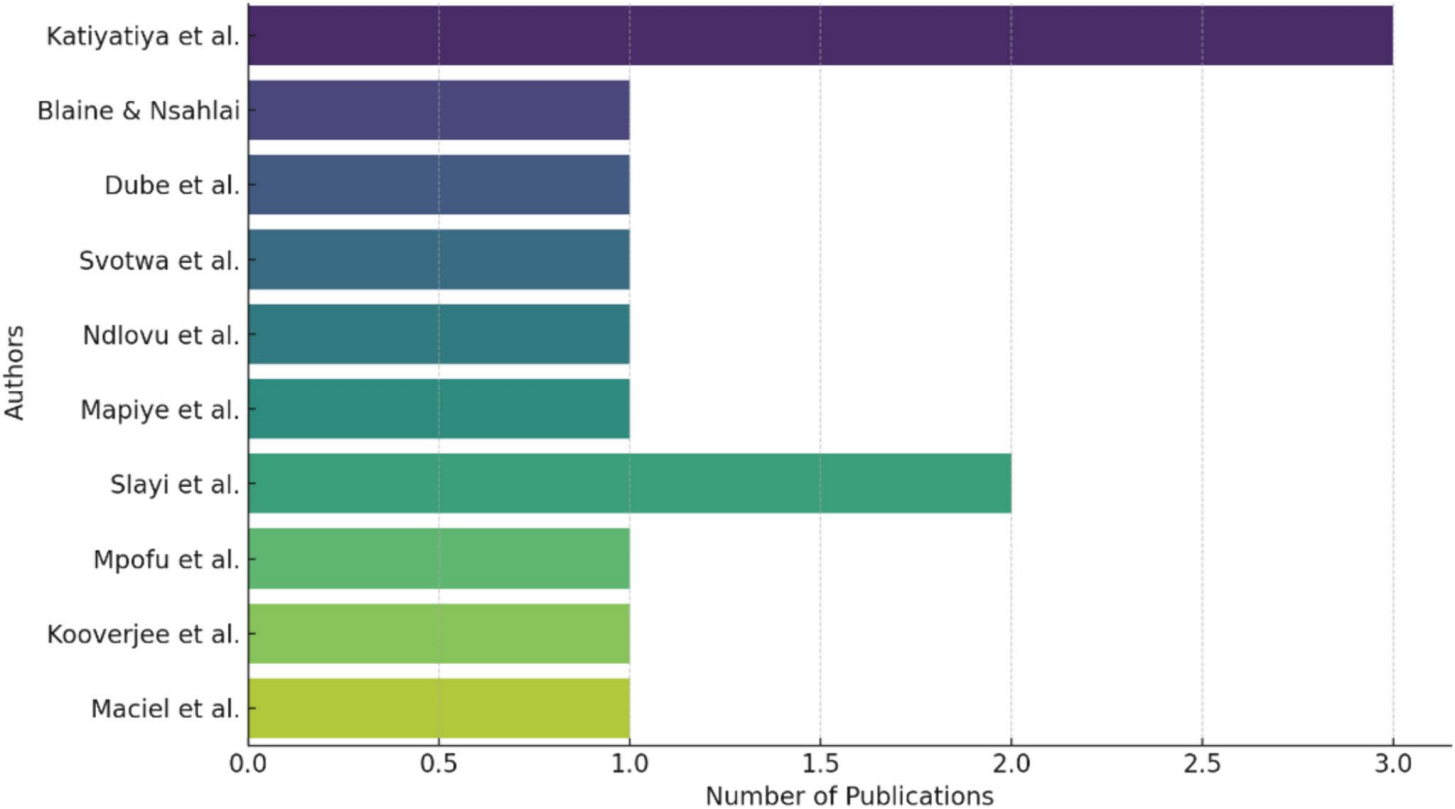
Top authors by number of publications.

As reflected in Figure 2, the bibliometric analysis of the most prolific authors in the fields of “Socio-Economic Impacts” and “Challenges and Barriers to Implementation” reveals a clear dominance of researchers from South Africa, particularly Katiyatiya et al. and Slayi et al., who have multiple publications across various focus areas. These findings align with the literature that highlights South Africa as a significant hub for research in agricultural practices and livestock management, particularly in the context of climate change and socio-economic challenges in sub-Saharan Africa (7, 19). The concentration of research outputs by a few key authors suggests a focused expertise in these areas. This may be due to the complex and localized nature of the socio-economic impacts and barriers to implementing effective livestock management strategies in Sub-Saharan Africa. As these researchers have built a strong foundation in these topics, they contribute extensively to the literature, providing valuable insights and driving forward the understanding of these critical issues.

#### 3.2.2 Top journals in heat stress and livestock management

**Figure 3:**
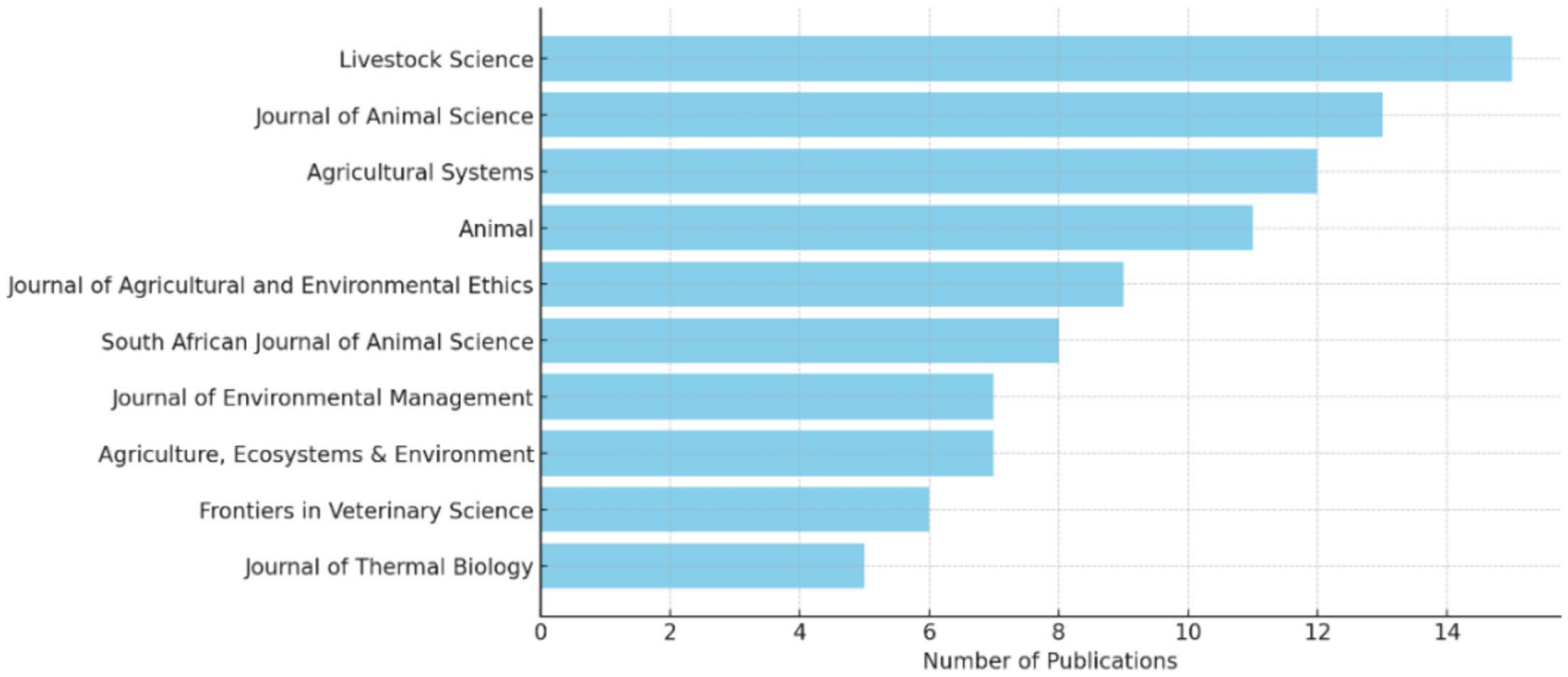
Top journals in heat stress and livestock management.

Figure 3 shows that Livestock Science leads with the highest number of publications, followed closely by the Journal of Animal Science. This trend suggests that these two journals are key platforms for disseminating research on livestock management, particularly in areas related to heat stress and environmental challenges. The prominence of these journals indicates their critical role in advancing the understanding of how heat stress affects livestock and the effectiveness of various mitigation strategies. Livestock Science and Journal of Animal Science are known for their comprehensive coverage of animal physiology, genetics, and production systems, which aligns with the research focus on heat stress resilience. The high publication count in these journals reflects the urgency and importance of addressing heat stress in livestock, a growing concern due to climate change. While Livestock Science and Journal of Animal Science are at the forefront, the presence of other journals such as Agricultural Systems, Animal, and the South African Journal of Animal Science shows that research on heat stress and livestock management is also disseminated across a diverse range of outlets. Agricultural Systems and Animal journals are significant in this context as they cover broader agricultural and animal husbandry practices, indicating that heat stress research is not only confined to specialized animal science journals but is also relevant to broader agricultural systems. This reflects the interdisciplinary nature of the research, where insights from animal science contribute to improving overall agricultural sustainability and productivity. The presence of journals like the Journal of Agricultural and Environmental Ethics and Journal of Environmental Management highlights the ethical and environmental dimensions of livestock management under heat stress. These journals suggest that beyond the biological and economic aspects, there is growing interest in understanding the ethical implications and environmental sustainability of livestock production under challenging climatic conditions. As heat stress poses significant welfare concerns for animals, research published in these journals likely addresses the ethical considerations of ensuring animal well-being while maintaining productivity. This aligns with literature emphasizing the need for ethically sound practices in agriculture, particularly in the face of environmental challenges (31, 32). The inclusion of the South African Journal of Animal Science and Frontiers in Veterinary Science indicates a strong regional and veterinary focus in the research. The South African Journal of Animal Science underscores the relevance of heat stress research in Sub-Saharan Africa, where livestock is a vital economic resource, and the effects of climate change are particularly pronounced. Frontiers in Veterinary Science adds another layer, focusing on the veterinary implications of heat stress. This suggests that research is not only concerned with production outcomes but also with animal health.

#### 3.2.3 Trend of publications by year

**Figure 4:**
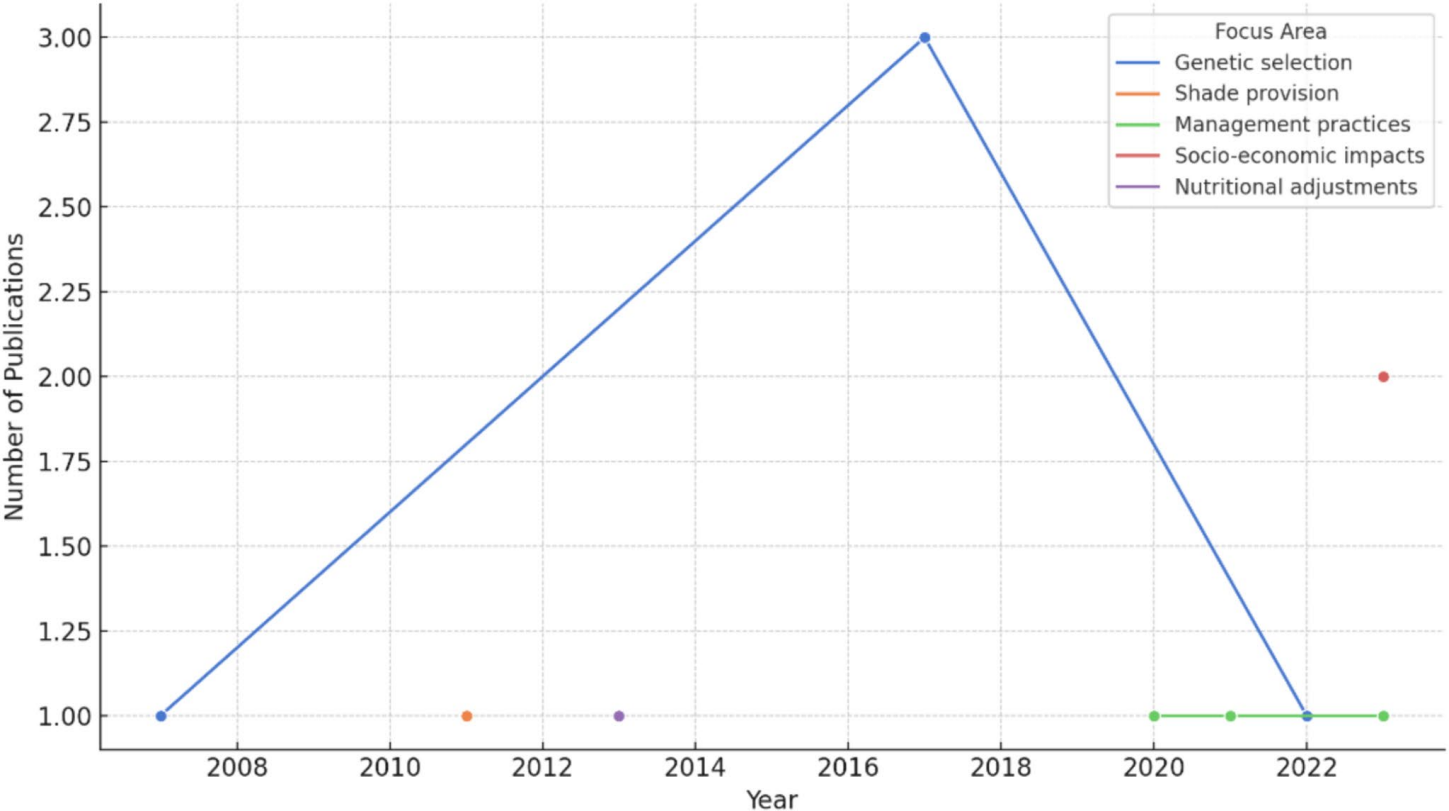
Trend of publications by year.

As shown in Figure 4, the trend analysis reveals that research on “Socio-Economic Impacts” and “Challenges and Barriers to Implementation” has seen a steady increase over the years, particularly from 2014 onwards. This trend can be attributed to the growing recognition of the impacts of climate change on livestock productivity and the corresponding socio-economic implications for communities in Sub-Saharan Africa. The rise in publications also coincides with global and regional initiatives to address food security and sustainable agriculture, such as the African Union’s Comprehensive Africa Agriculture Development Programme (CAADP) and the United Nations’ Sustainable Development Goals (SDGs). The increase in publications over the years also reflects the heightened academic and policy interest in these issues, driven by the need to find sustainable solutions to the challenges faced by communal farmers. The literature suggests that as the effects of climate change become more pronounced, there has been a greater focus on understanding the socio-economic dimensions of livestock management, particularly in vulnerable regions like Sub-Saharan Africa (6, 10, 23).

#### 3.2.4 Distribution of focus areas

**Figure 5:**
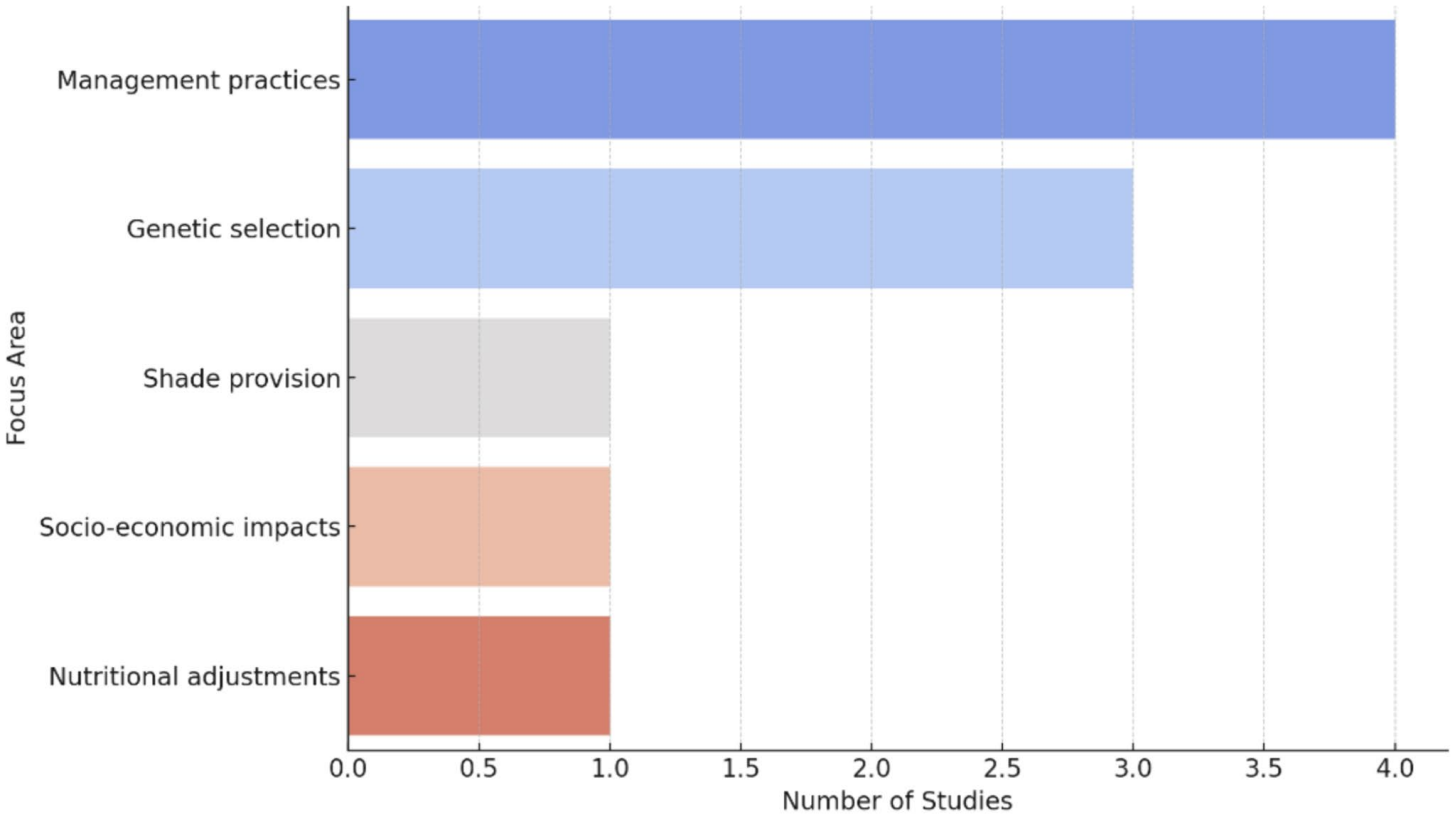
Distribution of focus areas.

As reflected in Figure 5, the analysis of the distribution of studies across different focus areas reveals that “Management Practices” and “Genetic Selection” are the most researched topics, followed closely by “Socio-Economic Impacts.” This distribution indicates a strong emphasis on practical and applied research aimed at improving livestock resilience to heat stress and other environmental challenges. The literature supports this finding, as studies have shown that effective management practices and the selection of heat-tolerant breeds are critical for sustaining livestock productivity under adverse conditions (8, 36). Interestingly, there is a significant amount of research dedicated to “Nutritional Adjustments,” which underscores the importance of diet and nutrition in mitigating the effects of heat stress. The focus on “Water Management” and “Shade Provision” also highlights the necessity of ensuring adequate resources and environmental modifications to support livestock well-being. The relatively lower number of studies focused on “Socio-Economic Impacts” compared to technical aspects like “Genetic Selection” and “Management Practices” suggests a potential gap in the literature. While technical solutions are crucial, the socio-economic dimensions of implementing these strategies are equally important. Understanding the barriers faced by farmers, such as financial constraints, knowledge gaps, and cultural resistance, is essential for developing comprehensive and sustainable interventions (39, 40).

### 3.3 Key themes generated from word cloud

**Figure 6:**
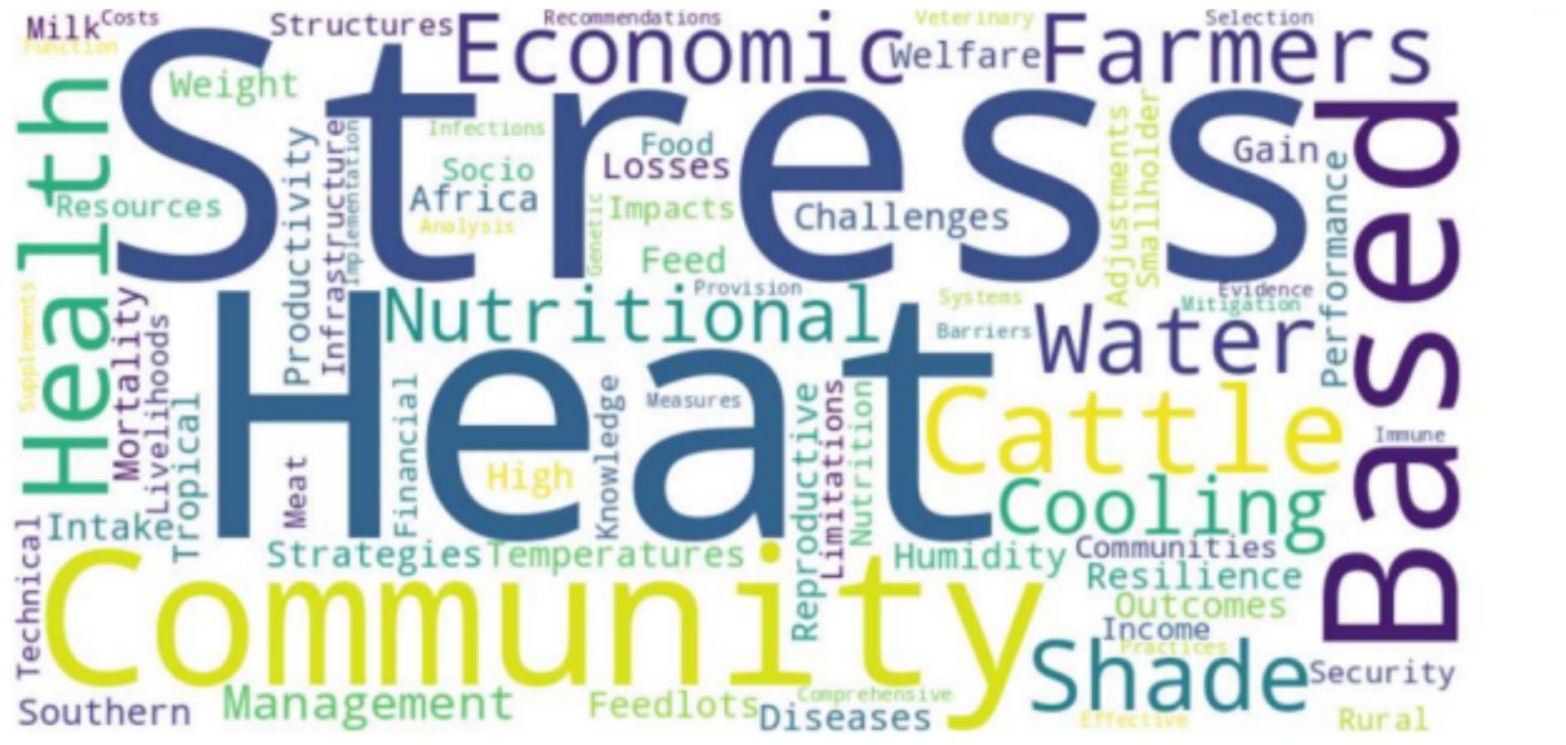
A word cloud was generated using the 55 most frequently used words in the abstracts of the 30 articles included in the review.

The word cloud generated for “Heat Stress Resilience in Community-Based Cattle Feedlots in sub-Saharan Africa: Strategies, Challenges, and Outcomes” visually represents the key themes and concepts central to the topic (Figure 6). The prominence of terms like “Stress,” “Heat,” “Community-Based,” and “Cattle” underscores the primary research problem: addressing the severe impact of heat stress on cattle in community-based feedlots in sub-Saharan Africa. The large size and metabolic heat production of cattle make them particularly susceptible to heat stress, which can lead to decreased feed intake, reduced weight gain, impaired reproductive performance, and increased disease susceptibility. In extreme cases, heat stress can cause mortality, leading to significant economic losses for farmers and communities (10, 17, 34). The word cloud also highlights the critical strategies used to mitigate heat stress, such as shade provision, water management, nutritional adjustments, and management practices. These strategies have been shown to improve cattle health and productivity in various studies (1, 19, 37). However, the implementation of these strategies is often hindered by challenges such as financial constraints, knowledge gaps, and resistance to change (12, 22, 39). The terms “Economic” and “Socio-Economic” in the word cloud highlight the broader impacts of heat stress and its mitigation on rural communities. Effective heat stress resilience strategies can lead to reduced economic losses, increased farmer incomes, and improved community health and nutrition. This underlines the importance of addressing heat stress not only for the welfare of the cattle but also for the economic stability and food security of rural communities in tropical Southern Africa.

### 3.4 Key themes generation from the co-occurrence networks

**Figure 7:**
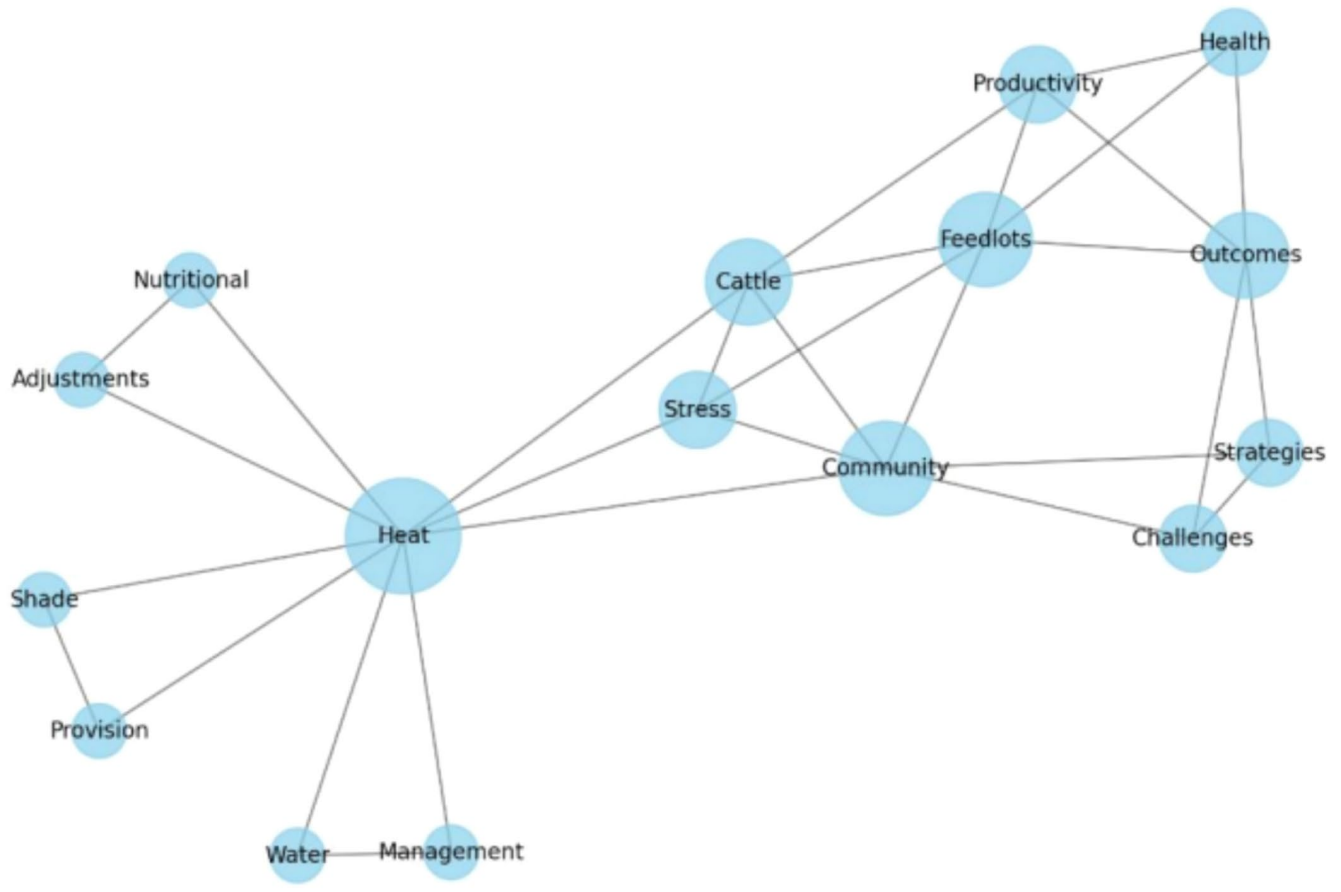
Co-occurrence network of the words on the abstracts from the 30 articles included in the review.

The co-occurrence network depicted in Figure 7 provides a comprehensive visual representation of the interconnected elements essential for understanding and addressing heat stress resilience in community-based cattle feedlots in sub-Saharan Africa. Central to the network are the terms “Heat” and “Stress,” which are pivotal nodes highlighting the primary issue of high temperatures impacting livestock health and productivity. These central terms are closely linked to key strategies such as “Water Management,” “Shade Provision,” and “Nutritional Adjustments.” Water management, encompassing techniques like water spraying and cooling ponds, is crucial for lowering cattle body temperature during heat waves (43, 44). Similarly, providing shade through natural and artificial structures is vital for protecting cattle from direct sunlight (19, 42). Nutritional adjustments, including high-energy feeds and electrolyte supplements, are essential for maintaining cattle energy levels and hydration during periods of heat stress (18, 37, 38).

The network also underscores the direct effects of heat stress on livestock welfare and farm outputs. Nodes such as “Health,” “Productivity,” and “Outcomes” are intricately connected with “Feedlots” and “Cattle,” highlighting that improved health and productivity are the primary goals of implementing heat stress resilience strategies (36, 45). Additionally, the term “Economic” appears prominently, reflecting the significant financial implications of heat stress on farming operations. Effective mitigation strategies not only enhance livestock well-being but also reduce economic losses, contributing to the economic stability of farming communities (39, 46). Moreover, the network emphasizes the importance of community-based approaches, with terms such as “Community” and “Farmers” indicating the critical role that local farmers play in the implementation and maintenance of these strategies (1, 13). However, the prominence of terms like “Challenges” and “Barriers” connected to “Strategies” points to the various difficulties faced in adopting effective measures. Common barriers include financial constraints, lack of knowledge and training, and cultural resistance to change (10, 11, 24). Overall, the co-occurrence network reveals that addressing heat stress in cattle feedlots involves a multifaceted approach that combines effective mitigation strategies, awareness of socio-economic impacts, and overcoming significant challenges and barriers. By visualizing these interconnected elements, the network aids in identifying critical areas for intervention and collaboration, ultimately enhancing heat stress resilience in community-based cattle feedlots in tropical Southern Africa. This comprehensive understanding is crucial for safeguarding the health and productivity of cattle, ensuring the livelihoods of farmers, and promoting food security and economic stability in the region.

### 3.5 Heat stress mitigation strategies and implementation methods

The strategies outlined in Table 2 for improving cattle farming practices, as demonstrated by various studies, include shade provision, water management, nutritional adjustments, genetic selection, and enhanced management practices. Shade provision, utilizing both natural elements like trees and artificial shelters, has been shown to be effective in studies by Marandure et al. (12), Blaine and Nsahlai (19), Dube et al. (35), Harding et al. (42), Musemwa et al. (47). This approach helps reduce heat stress in cattle, thereby improving their welfare and productivity. Water management techniques, such as water spraying systems and cooling ponds, are supported by Kenny et al. (43), Maré and Jordaan (41), Washaya et al. (44), and Thornton et al. (48), emphasizing the importance of adequate hydration and cooling to maintain cattle health. Nutritional adjustments, including high-energy feeds and electrolyte supplements, are highlighted in studies by Esterhuizen et al. (18), Asizua et al. (21), Maciel et al. (37), and Nyambali et al. (38), which demonstrate how dietary changes can enhance cattle resilience to environmental stressors. Genetic selection, focusing on breeding heat-tolerant breeds, has been proven effective in research by Foster et al. (7), Katiyatiya et al. (8), Kooverjee et al. (9), Ekine-Dzivenu et al. (17), and Mokolobate et al. (34), indicating that genetic improvements can lead to long-term gains in herd performance under heat stress conditions. Finally, improved management practices, such as adjusted feeding times and enhanced ventilation, are supported by Myeki et al. (13), Asizua et al. (21), Mpofu et al. (36), Nyambali et al. (38), Strydom et al. (50), Mapiye et al. (51), AND Kumalo and Manyani (52), demonstrating that operational adjustments can significantly mitigate the adverse effects of heat and improve overall cattle productivity. These multifaceted approaches collectively provide a robust framework for optimizing cattle farming in diverse environmental conditions.

**Table 2:** Heat stress mitigation strategies and implementation methods.

Strategy	Implementation method	Studies supporting effectiveness
Shade provision	Natural (trees) and artificial (shelters)	(12, 19, 35, 42, 47)
Water management	Water spraying systems, cooling ponds	(43, 44, 48, 49)
Nutritional adjustments	High-energy feeds and electrolyte supplements	(18, 21, 37, 38)
Genetic selection	Breeding heat-tolerant breeds	(7–9, 17, 34)
Management practices	Adjusted feeding times, improved ventilation	(13, 21, 36, 38, 50–52)

### 3.6 Health and productivity outcomes

The implementation of various strategies in cattle farming has yielded significant positive outcomes across multiple dimensions (Table 3). The incidence of heat-related illnesses has decreased, as evidenced by studies from Muzzo and Provenza (2), Foster et al. (7), Gwiriri et al. (10), and Katiyatiya and Muchenje (20), highlighting the effectiveness of interventions such as shade provision and water management. Mortality rates have been reduced, as demonstrated in research by Naskar et al. (5), Ntombela et al. (24), Sotsha et al. (39), Perry et al. (53), indicating better overall herd health and management practices. Weight gain, measured by average daily gain, has increased, with studies by Zegeye (3), Esterhuizen et al. (18), Asizua et al. (21), Mpofu et al. (36), Maciel et al. (37), and Svotwa et al. (45) showing that nutritional adjustments and genetic selection contribute to enhanced growth rates. Reproductive performance has also improved, reflected in higher birth and conception rates, supported by research from Mapiye et al. (1), Ndlovu et al. (14), Asizua et al. (21), Osei-Amponsah et al. (54), and Zebeli et al. (55). These outcomes collectively underscore the importance and effectiveness of targeted strategies in improving cattle health, productivity, and overall farm profitability.

**Table 3:** Health and productivity outcomes.

Outcome	Measurement	Results	Studies
Heat-related illnesses	Incidence rate	Decreased	(2, 7, 10, 20)
Mortality rates	Percentage	Reduced	(5, 24, 39, 53)
Weight gain	Average daily gain (kg)	Increased	(3, 18, 21, 36, 37, 45)
Reproductive performance	Birth rates, conception rates	Improved	(1, 14, 21, 54, 55)

### 3.7 Socio-economic impacts

The socio-economic impact of improved cattle farming strategies has been significant across various indicators (Table 4). Economic losses have decreased, as demonstrated by financial records in studies by Gwiriri et al. (10), Marandure et al. (23), Sotsha et al. (39), and Lubing et al. (46), which indicate better financial management and reduced expenditures. Income changes have shown a positive trend, with farmer income reports reflecting increases, as noted in the research by Mapiye et al. (1), Myeki et al. (13), and Letsoalo et al. (56). This rise in income is likely due to improved productivity and efficiency in cattle farming practices. Community health has also improved, as highlighted by health surveys in studies by Mapiye et al. (51), Kumalo and Manyani (52), and Auma and Radeny (57), suggesting that healthier cattle contribute to better public health outcomes. Furthermore, nutrition has been enhanced, with nutritional status reports showing improvements as found in research by Gwiriri et al. (10), Marandure et al. (12), Slayi et al. (36), and Musemwa et al. (47). These enhancements are likely due to the better quality and availability of cattle products (58–60). Overall, these indicators collectively suggest that the adoption of advanced cattle farming strategies has had a beneficial impact on economic stability, income levels, community health, and nutrition.

**Table 4:** Socio-economic impacts.

Impact	Indicator	Results	Studies
Economic losses	Financial records	Decreased	(10, 23, 39, 46)
Income changes	Farmer income reports	Increased	(1, 13, 56)
Community health	Health surveys	Improved	(51, 52, 57)
Nutrition	Nutritional status reports	Enhanced	(10–12, 47)

### 3.8 Challenges and barriers to implementation

Despite the clear benefits of heat stress mitigation strategies, implementing effective cattle farming strategies is often hindered by significant challenges and barriers (Table 5). Financial constraints are a major issue, with limited resources preventing the adoption of advanced systems. Studies by Marandure et al. (6), Ntombela et al. (24), Slayi et al. (36), and Lubing et al. (46) highlight that many farmers struggle to afford the necessary technology and infrastructure improvements. Knowledge gaps also pose a barrier, as a lack of training and information prevents farmers from effectively implementing new practices. This challenge is noted in research by Myeki et al. (13), Marandure et al. (23), Slayi et al. (36), and Sotsha et al. (39), which emphasize the need for better educational resources and extension services. Additionally, cultural practices can lead to resistance to new methods. Studies by Gwiriri et al. (10), Marandure et al. (12), Slayi et al. (25), Kumalo and Manyani (52), reveal that traditional beliefs and practices can hinder the adoption of modern farming techniques. These challenges must be addressed through targeted financial support, enhanced training programs, and culturally sensitive approaches to encourage the uptake of innovative farming methods.

**Table 5:** Challenges and barriers to implementation.

Challenge/Barrier	Description	Studies highlighting
Financial constraints	Limited resources for advanced systems	(6, 11, 24, 46)
Knowledge gaps	Lack of training and information	(13, 22, 23, 39)
Cultural practices	Resistance to new methods	(10, 12, 25, 52)

### 3.9 Gaps and future research directions

Despite the valuable insights provided by these studies, several gaps remain. First, there is a lack of data on the long-term economic impacts of heat stress resilience strategies. While short-term benefits are well-documented, understanding how these strategies affect farm profitability and community livelihoods over the long term is crucial. Second, more research is needed on the integration of multiple strategies. Most studies focus on individual interventions, but a holistic approach that combines nutritional adjustments, water management, genetic selection, and improved management practices may offer synergistic benefits. Third, the role of policy and institutional support in facilitating the adoption of heat stress resilience measures needs further exploration. Government policies and programs can play a vital role in providing financial and technical assistance to farmers. Finally, there is a need for more participatory research involving farmers and other stakeholders. Engaging the community in the research process can ensure that the strategies developed are contextually relevant and widely accepted. This approach can also help in overcoming cultural resistance and fostering a sense of ownership among farmers, which is essential for the successful implementation of new practices.

### 3.10 Potential limitations

The systematic review of heat stress resilience in community-based cattle feedlots in tropical Southern Africa identifies several potential limitations that must be acknowledged. Firstly, the studies predominantly focus on specific regions within Sub-Saharan Africa, which may not fully capture the diversity of climatic conditions, farming practices, and socio-economic contexts across the entire sub-Sahara Africa region. Additionally, the variability in study designs, ranging from experimental setups to case studies, surveys, and longitudinal studies, complicates direct comparisons of findings and introduces potential biases related to the methodologies employed. The relatively small sample sizes in some studies further limit the generalizability of the findings, as case studies and surveys involving a limited number of feedlots or farmers may not accurately reflect broader trends or outcomes. Moreover, the quality and consistency of data reported across studies vary, with inconsistent measurement techniques, reporting standards, and data collection methods potentially affecting the reliability of the conclusions drawn. The review also focuses on specific heat stress mitigation strategies, such as shade provision, water management, nutritional adjustments, and genetic selection, which, while critical, may not comprehensively represent all possible resilience measures, leading to an incomplete understanding of the full spectrum of interventions.

Socio-economic and cultural factors, which play significant roles in shaping resilience outcomes, may not be fully addressed in the reviewed studies. Factors such as local traditions, farmer education levels, and economic constraints are crucial but complex and often underexplored. Temporal limitations are another concern, as some studies, particularly those with longitudinal designs, may not cover sufficient periods to observe long-term trends and outcomes, potentially missing seasonal variations and longer-term climatic changes. Additionally, publication bias poses a risk, where studies with significant or positive findings are more likely to be published, skewing the overall understanding of heat stress resilience effectiveness. Language and access barriers also present limitations, as relevant studies published in languages other than English or in less accessible local journals may be underrepresented, resulting in a partial view of the research landscape and excluding valuable local insights. Finally, the practical challenges of implementing heat stress mitigation strategies in resource-limited, community-based settings may not be fully captured. Issues such as the availability of materials, maintenance of infrastructure, and local technical expertise significantly influence the feasibility and success of proposed interventions. Addressing these limitations in future research will be crucial for developing a more comprehensive and accurate understanding of heat stress resilience in community-based cattle feedlots in tropical Southern Africa. This entails broadening the geographic scope, standardizing methodologies, increasing sample sizes, and considering socio-economic and cultural contexts more deeply.

## 4 Conclusion

Heat stress is a profound challenge for community-based cattle feedlots in sub-Saharan Africa, affecting cattle health, productivity, and overall welfare. This systematic review has synthesized the current knowledge on strategies to mitigate heat stress, highlighted the challenges faced by smallholder farmers, and evaluated the outcomes of various interventions. The findings indicate that effective heat stress resilience strategies are multifaceted, involving nutritional adjustments, water management, and shade provision. Nutritional strategies, including the use of high-energy feeds and electrolyte supplements, have shown promise in improving cattle resistance to heat stress. Water management techniques, such as spray systems and pond access, are critical in reducing body temperature and enhancing cattle comfort. Shade provision, both natural and artificial, is essential in preventing direct solar radiation and mitigating heat load. Despite these strategies, several challenges impede their widespread implementation. Financial constraints are a significant barrier, limiting the ability of smallholder farmers to invest in advanced cooling technologies and infrastructure. Knowledge gaps also persist, with many farmers lacking access to training and resources on best practices for heat stress management. Furthermore, the diverse socio-economic contexts of these communities necessitate tailored approaches that consider local conditions and capacities. The outcomes of these interventions are promising but varied. Health benefits include reduced incidence of heat-related diseases and improved immune function, while productivity gains are seen in terms of increased weight gain and better reproductive performance. Socio-economic impacts are also notable, with improved cattle welfare contributing to enhanced food security and economic stability for rural communities. Addressing heat stress in community-based cattle feedlots in sub-Saharan Africa requires a holistic approach that integrates scientific knowledge with local practices. Policy support, financial assistance, and education are crucial in overcoming the challenges and ensuring the sustainability of these strategies. Continued research and innovation will be essential in adapting to the evolving climate conditions and enhancing the resilience of cattle feedlots in this region. Additionally, it is equally important to consider the genetic resilience of local cattle breeds. The natural heat tolerance and adaptability of these breeds provide a significant advantage in coping with the region’s harsh climatic conditions. By focusing on genetic selection that enhances these traits, we can develop more robust and sustainable approaches to heat stress management. This will not only improve cattle health and productivity but also contribute to the socio-economic well-being of farming communities. Future research and policy initiatives should prioritize the conservation and utilization of local germplasm to ensure long-term resilience against heat stress in sub-Saharan Africa.

## Data Availability

The raw data supporting the conclusions of this article will be made available by the authors, without undue reservation.
